# Supplementary figures and images for: A systemic cell cycle block impacts stage-specific histone modification profiles during Xenopus embryogenesis
Source: PLoS Biol. 2021 Sep 7;19(9):e3001377. doi: 10.1371/journal.pbio.3001377 (PMC8535184; doi:10.1371/journal.pbio.3001377)

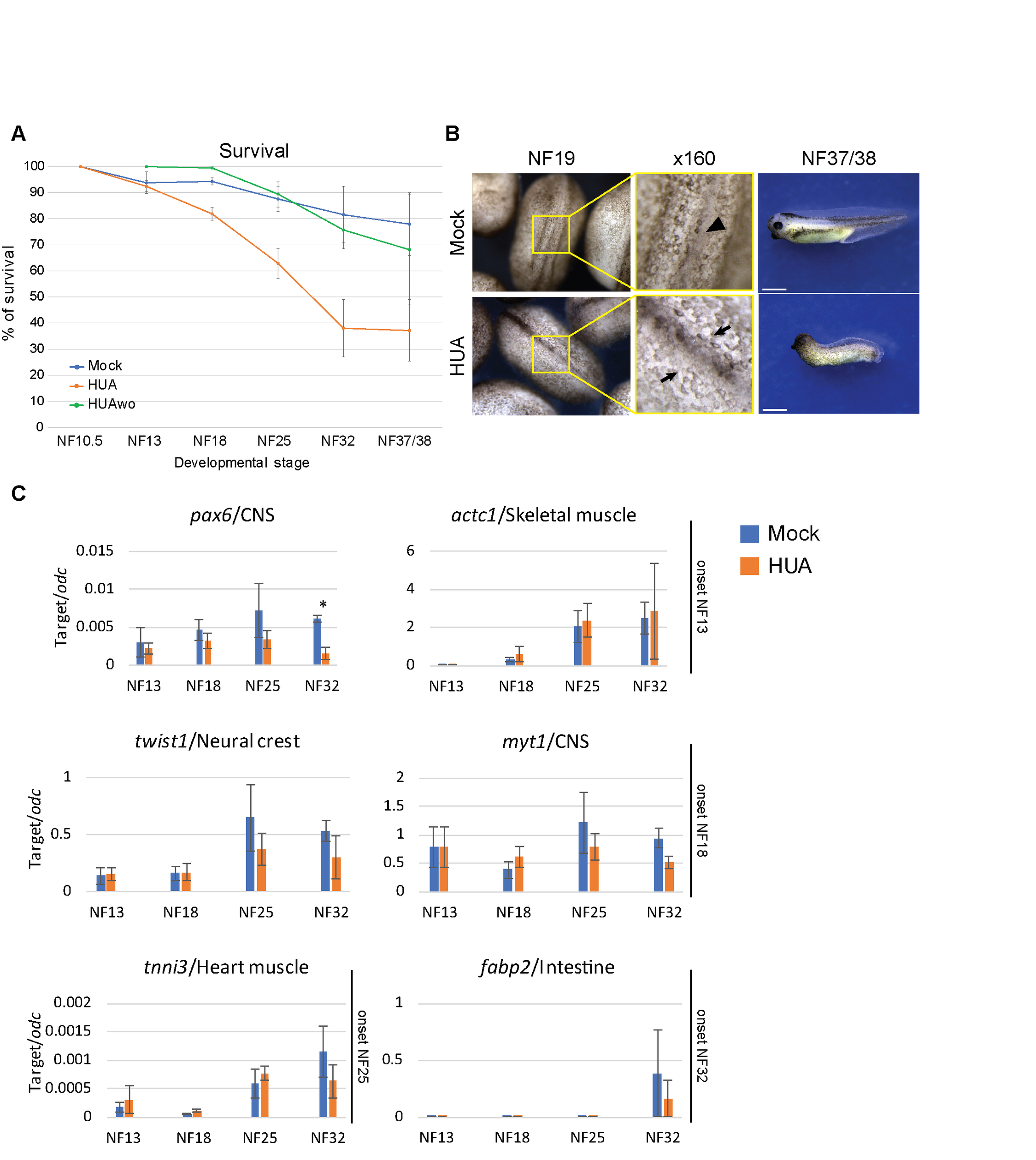

Supplement: S1 Fig — (A) Embryonic survival curves under Mock, HUA, or HUAwo condition. Data from n ≥ 3 biological replicates/condition; mean ± SEM. The individual quantitative observations can be found in S5 Data (sheet 1). (B) The first morphological effect of HUA treatment is apparent at stage NF19 as a delay in neural tube closure (in mock, black arrowhead points to the dorsal midline; in HUA, 2 black arrows point to separate neural folds). Under higher magnification, HUA embryos contain larger cells. After hatching (stage NF37/38), HUA-treated embryos lack tails, have reduced eyes and malformed fins, and are largely deficient in melanocytes. (C) Comparison of temporal expression profiles for selected marker genes in Mock and HUA conditions by qRT/PCR, normalized to odc mRNA. Genes are grouped according to their activation time point. N = 3 biological replicates/condition; mean ± SEM. Significant difference was detected only in case of pax6 expression level at NF32 stage (Student t test [two-tailed, paired]; * p < 0.05). No other significant differences were detected between the 2 conditions. The individual quantitative observations can be found in S5 Data (sheet 2). CNS, central nervous system; HUA, hydroxyurea and aphidicolin; HUAwo, HUA washout; qRT/PCR, quantitative reverse transcription polymerase chain reaction. (TIF) [file pbio.3001377.s001.tif]

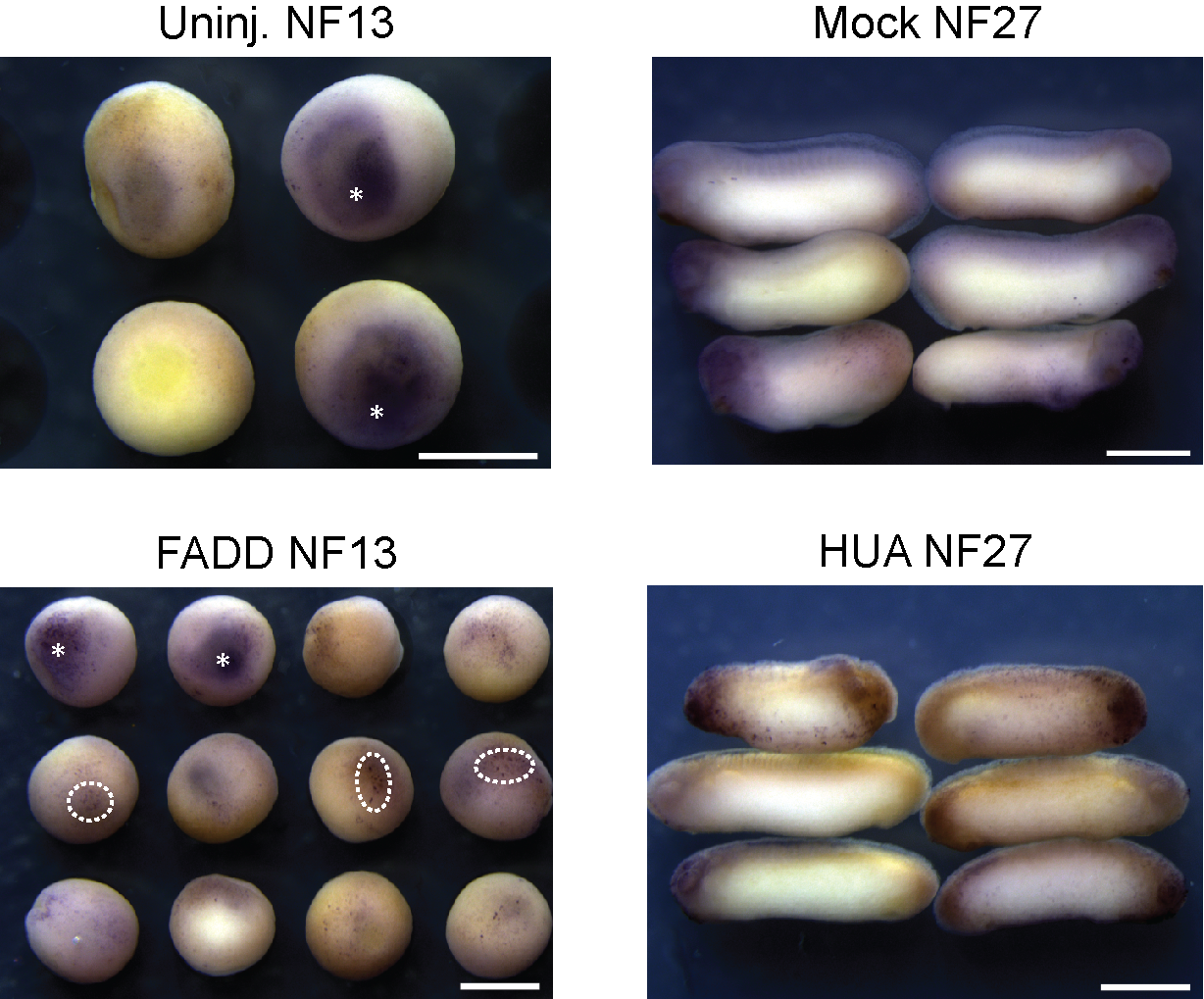

Supplement: S2 Fig — Wild-type embryos were injected with FADD apoptosis inducing plasmid in one blastomere at 4-cell stage as a positive control. FADD-injected and FADD-uninjected wild-type embryos together with Mock and HUA embryos were ICC stained against activated cas-3. Signal from cas-3 staining can be observed on the skin of the embryos as small blue dots; dashed ovals highlight FADD-induced cas-3 staining; white asterisks indicate blastocoel background staining, scale bar: 1 mm. HUA embryos do not demonstrate an increased level of apoptosis compared to Mock siblings. cas-3, caspase-3; FADD, Fas-associated protein with death domain; HUA, hydroxyurea and aphidicolin; ICC, immunocytochemical staining. (TIF) [file pbio.3001377.s002.tif]

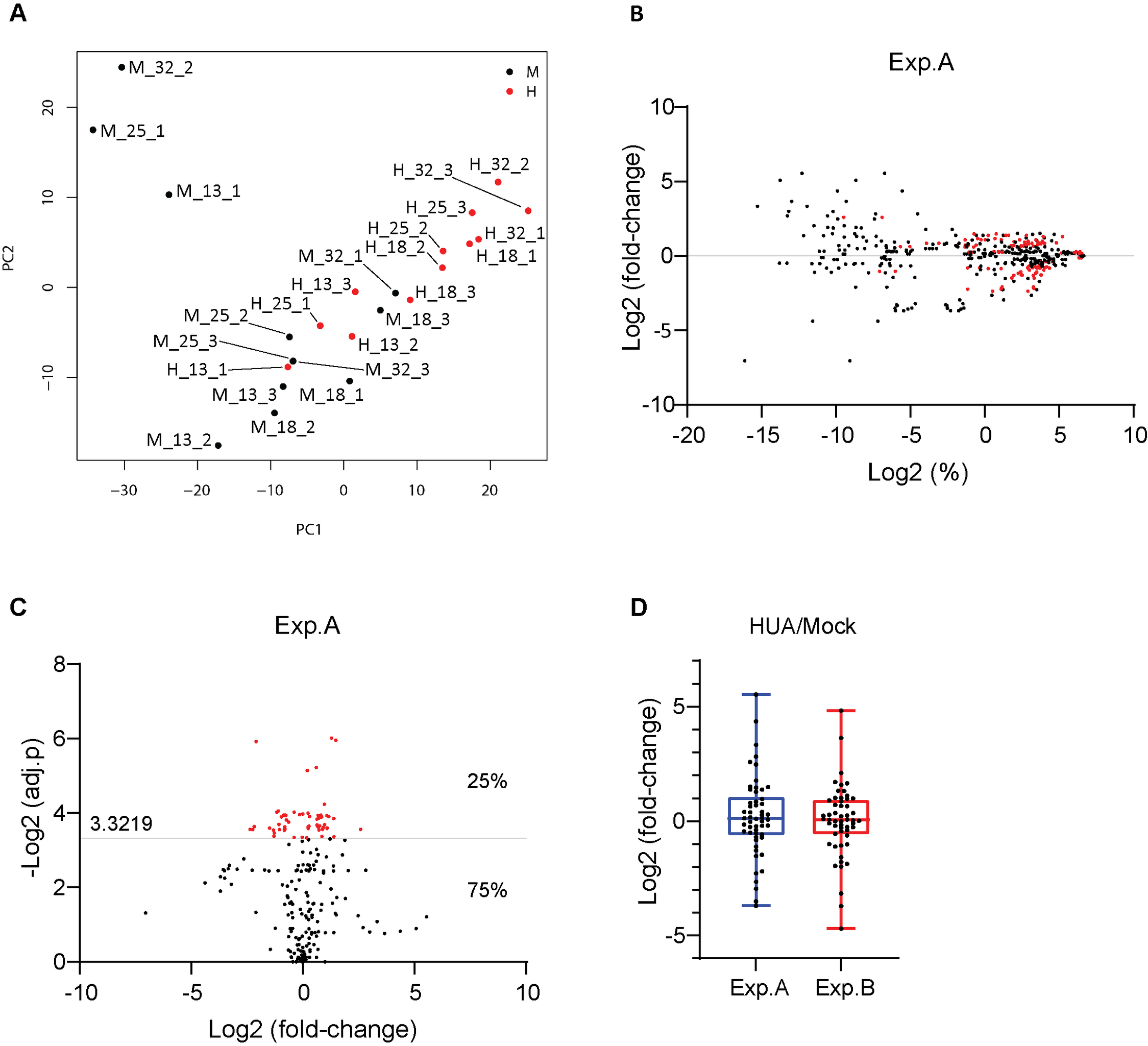

Supplement: S3 Fig — (A) PCA for Mock- and HUA-treated HPMs (Exp. A). Each data point represents 64 modification states, measured by LC–MS/MS in PRM mode, with absolute abundance calculated with R10 spiketide normalization. Mock and HUA data sets are partially separated, with younger HUA samples intermingling with older Mock samples. (B) MA-plot detailing the distribution of Log2 transformed relative histone PTM abundance [Log2(%)] over Log2 transformed fold-change between HUA and Mock conditions [Log2(fold-change)] (Exp. A). Histone PTMs different between HUA and Mock conditions with adj.p < 0.1 (Benjamini–Hochberg procedure) highlighted in red. Lower abundant modifications tend to have larger variability. (C) Volcano plot shows the distribution of Log2 transformed fold-change [Log2(fold-change)] over negative Log2 transformed adj.p-value [−Log2(adj.p)] between HUA and Mock conditions (Exp. A). Baseline in gray: 3.3219 as −Log2(0.1). Histone PTMs different between HUA and Mock with adj.p < 0.1 (Benjamini−Hochberg procedure) are above the baseline, highlighted in red. One-quarter of the analyzed histone PTMs is significantly different between Mock and HUA conditions. (D) Boxplot indicates the distribution of Log2 transformed fold-changes [Log2(fold-change)] between HUA and Mock conditions in Exp. A and Exp. B. Median is shown. The results from Exp. A and Exp. B are similar. The direct comparison of relative abundance of the histone PTMs is shown in S6 Fig. The individual quantitative observations can be found in S6 Data. HMP, histone modification profile; HUA, hydroxyurea and aphidicolin; LC–MS/MS, liquid chromatography–tandem mass spectrometry; PCA, principal component analysis; PRM, parallel reaction monitoring; PTM, posttranslational modification of histone. (TIF) [file pbio.3001377.s003.tif]

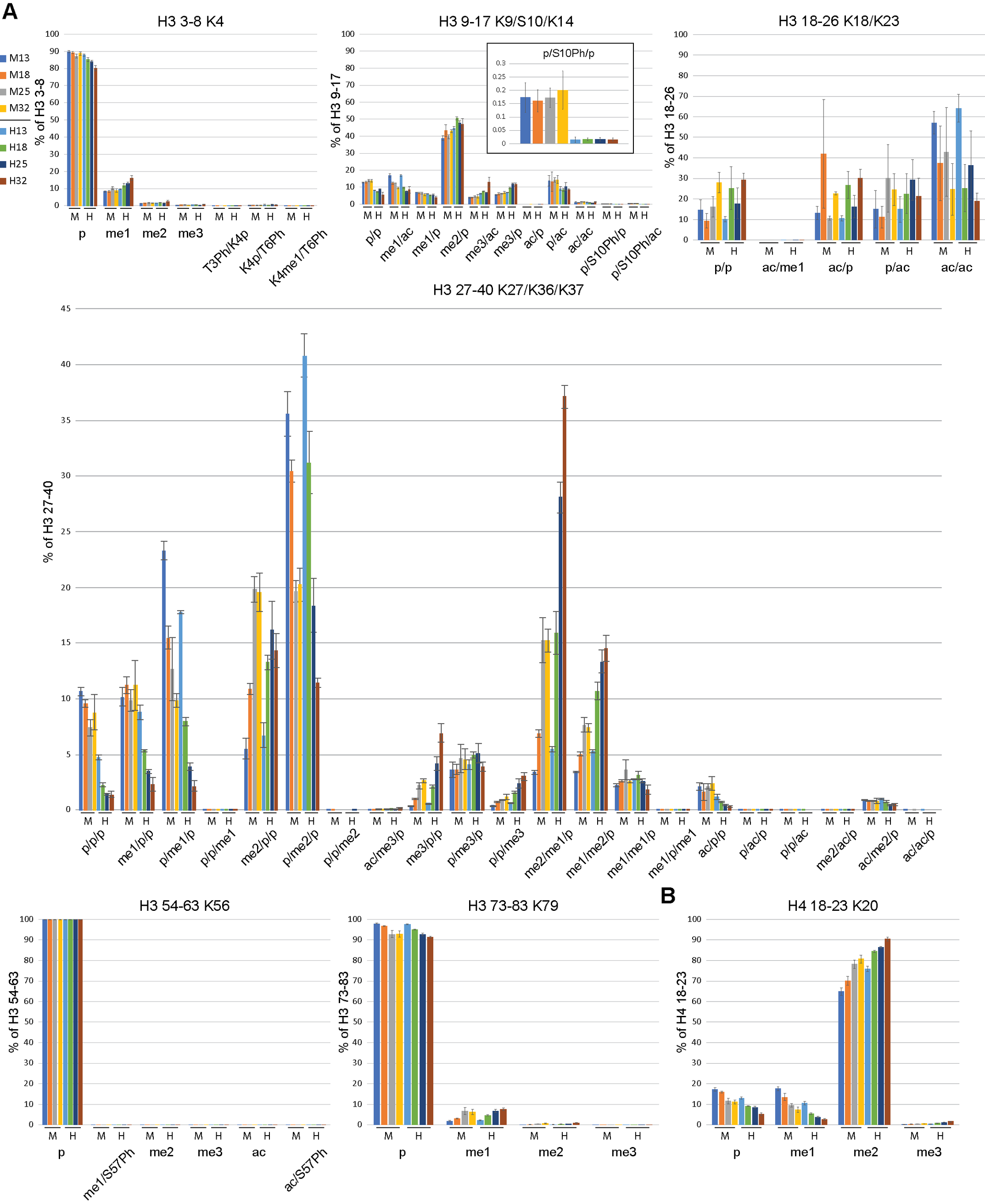

Supplement: S4 Fig — (A) Individual relative histone PTM distribution for histone H3. Data are first normalized to R10 spiketide signals, then added up to 100% for all modification states measured for each specific tryptic peptide, from which the relative contribution of each state is then calculated. The insert in H3 9–17 K9/S10/K14 plot shows a zoom-in for the H3S10Ph mark. (B) Individual relative histone PTM distribution for histone H4. N = 3 biological replicates/condition; mean ± SEM. “p,” propionylated (naturally unmodified). The individual quantitative observations can be found in S5 Table. ac, acetylation; HUA, hydroxyurea and aphidicolin; me, methylation; Ph, phosphorylation; PTM, posttranslational modification of histone. (TIF) [file pbio.3001377.s004.tif]

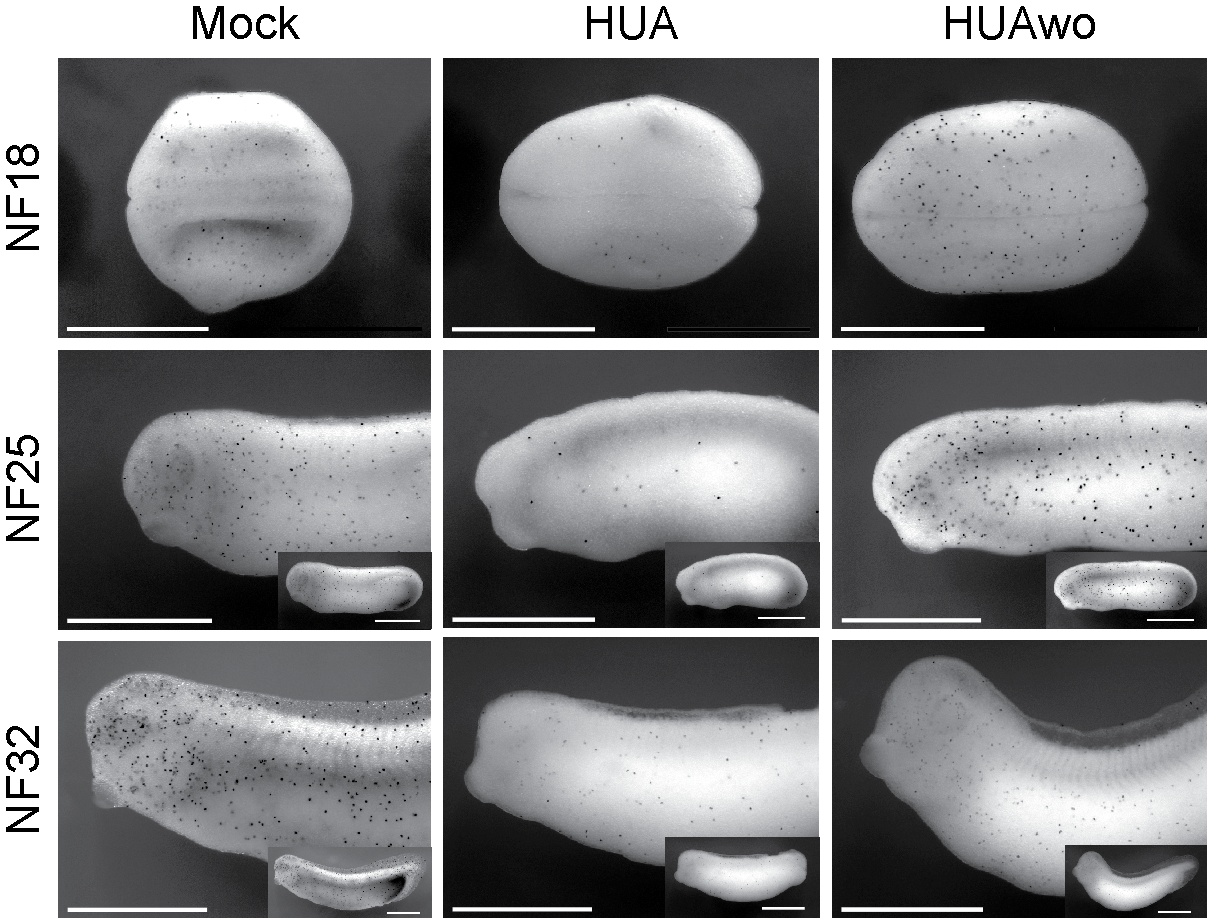

Supplement: S5 Fig — ICC for the mitotic histone mark H3S10Ph at indicated stages. Mitotic cells are marked by black dots. Elongated, older embryos are recorded as anterior halves, i.e., at the same magnification as younger stages, and in whole mount views as inserts. Scale bars: 1 mm. N ≥ 3 biological replicates/condition. HUAwo, HUA washout; ICC, immunocytochemical staining. (TIF) [file pbio.3001377.s005.tif]

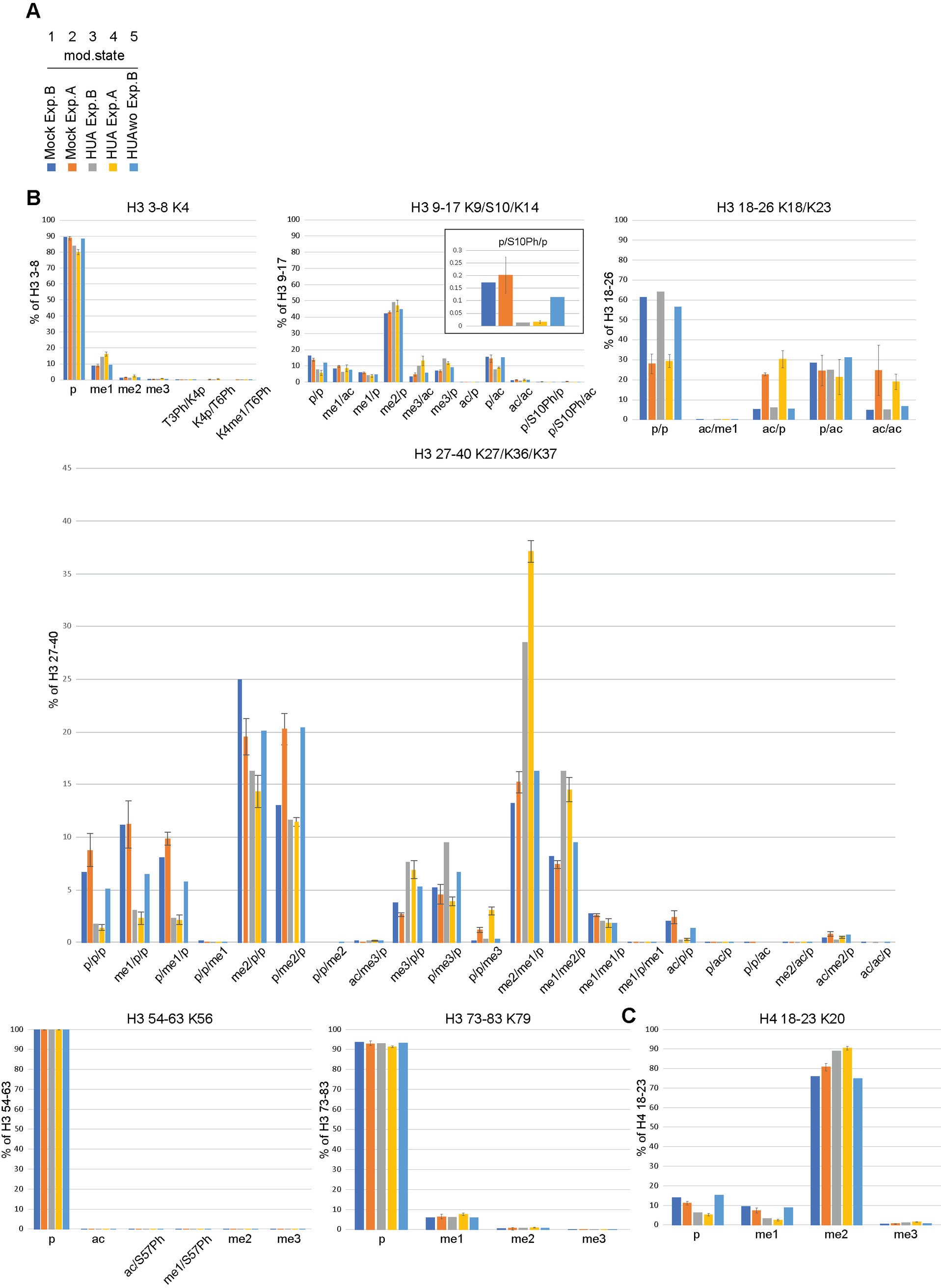

Supplement: S6 Fig — Relative histone PTMs abundance was calculated as described in Materials and methods. The insert in H3 9–17 K9/S10/K14 plot shows a zoom-in for the H3S10Ph mark. (A) Color coding of sample types. Panels (B) and (C): individual relative histone PTM distributions for histone H3 and H4, respectively. “p,” propionylated (naturally unmodified). The individual quantitative observations can be found in S6 Table. ac, acetylation; me, methylation; Ph, phosphorylation; PTM, posttranslational modification of histone. (TIF) [file pbio.3001377.s006.tif]

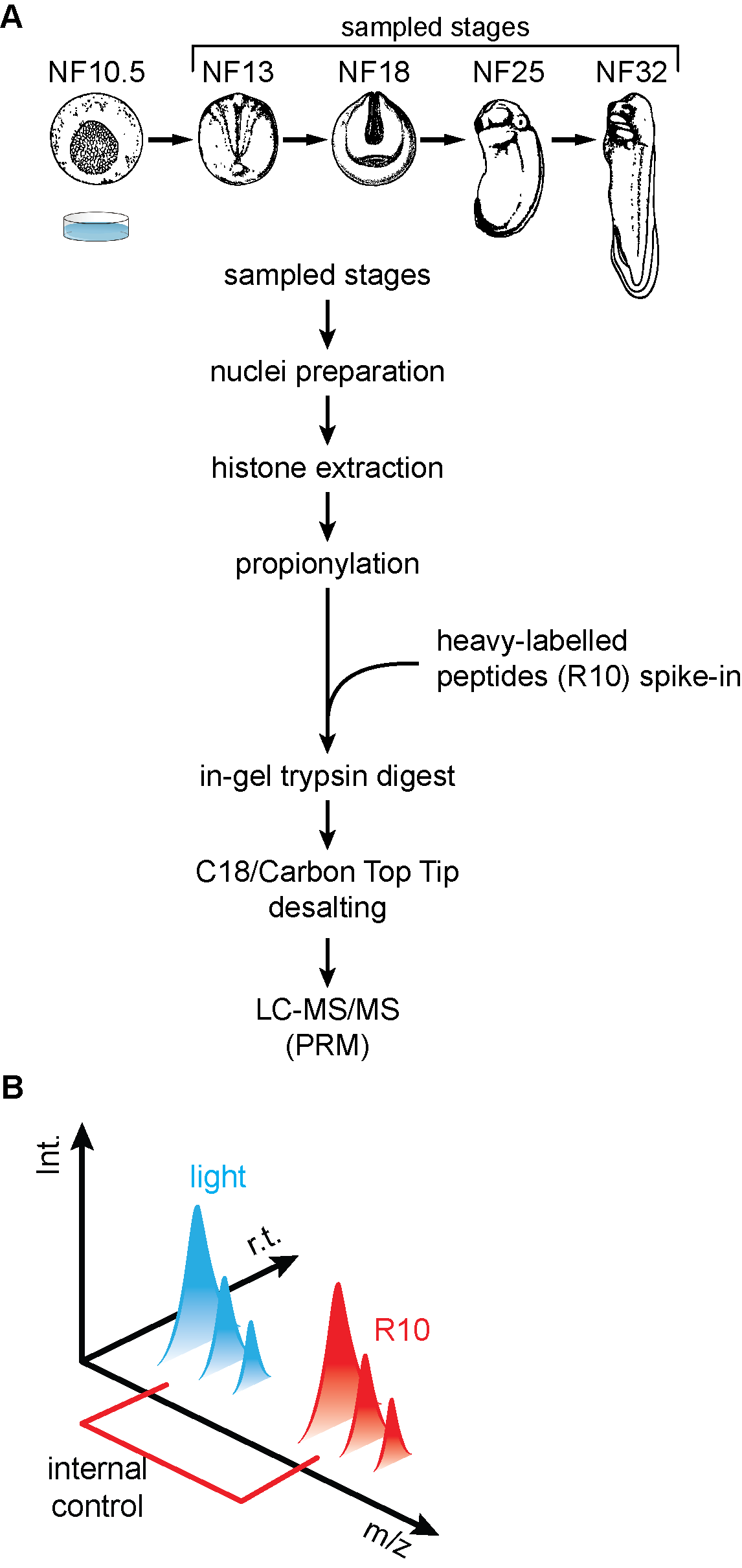

Supplement: S7 Fig — (A) Pipeline of mass spectrometry analysis of histone modifications from Xenopus laevis. Bulk histones are isolated from purified nuclei of embryos from 4 sampled stages (see Fig 1) by acidic extraction and SDS-PAGE. Propionylation blocks all endogenously unmodified and monomethylated lysine residues from being cleaved in the subsequent trypsin digest, thereby creating an optimized peptide pool for Mass Spec analysis. Due to this step, naturally unmodified lysine residues are labeled as “p,” tryptic peptides which have no modification states indicated as “un.” After propionylation, but before trypsin digest, we add to each sample a so-called R10 library (S2 Table), which consists of isotopically heavy-labeled arginine peptides (R10). The individual R10 peptides are mixed in equimolar concentration and mimic 64 histone H3 and H4 modification states. These isotopically heavy-labeled peptides serve as an internal and intersample control, allowing to minimize technical variations and to quantitate abundance of histone modification states on the absolute scale. (B) Representation of the R10 spike-in peptide control. Each of the analyzed endogenous histone modification states has a synthetized R10 peptide analog. Due to the same chemical properties, endogenous tryptic peptides and their R10 spiketide analogs elute at the same RT; however, they can be distinguished based on the mass to charge (m/z) ratio. Additionally, R10 spiketides help with peak identification based on RT and detail fragmentation spectra for isobaric peptides. LC–MS/MS, liquid chromatography–tandem mass spectrometry; PRM, parallel reaction monitoring; RT, retention time. (TIF) [file pbio.3001377.s007.tif]
