# Supplementary material for: A systemic cell cycle block impacts stage-specific histone modification profiles during Xenopus embryogenesis
Source: PLoS Biol. 2021 Sep 7;19(9):e3001377. doi: 10.1371/journal.pbio.3001377 (PMC8535184; doi:10.1371/journal.pbio.3001377)
Supplement: Attachment — Submitted filename: Response to Reviewers.docx [file pbio.3001377.s024.docx]

**Response to Reviewers**

We want to thank both reviewers for their constructive suggestions and for raising several insightful points!

In our revision, we:

- discuss how cellular stress, DNA-damage and cell size could contribute to the alterations in stage-specific histone modification profiles.
- present p-values to assess statistical significance of the observed fold-change in PTMs between cycling and arrested chromatin.
- add new statistical analysis and data visualisation found in Figures 4, 6, S3 panels B-D, S4, S6, and in supplementary Table S5.
- perform additional experiments to resolve a potential discrepancy for H3T3Ph abundance.
- revised the text carefully to remove ambiguities and provide a most accurate description of our results and conclusions.

In blue – reviewers’ original comments

In black – answers to the comments

## Reviewer 1:

This is an interesting manuscript that investigates the relationship between chromatin modifications and proliferation in the Xenopus embryos from gastrula to tailbud stages. They inhibit DNA replication with a combination of hydroxyurea and aphidicolin and then assess morphology and bulk chromatin modifications by mass spec. Consistent with previous work from others, embryos undergo mostly normal morphogenesis through early tailbud, aside from some differences in the size of eye and tail precursors and a relative enlargement of individual cell sizes. The most novel and interesting aspect of the work is the definition of a "stage-specific histone modification profile" (HMP) for each stage of development; these HMPs appear to be distinct from one another and are a novel method of characterizing global histone modifications in development. The authors show that exposure to HUA dramatically alters the HMPs at each stage and that this can be reversed by washing out the HUA.

Overall, this is an interesting and thoughtful body of work, and I do not have experimental concerns, but I do have suggestions for clarification or revision to the text.

### Comment 1.

The authors at times assume causality when this is not supported by the data. For example, the statement "These results indicate that replicational dilution of histone marks has a strong impact on developmental chromatin maturation" is not yet supported by the data. They have not shown that proliferation or cell cycle progression directly affects chromatin state. They show that the HMP varies with stage and that replication inhibitors alter these profiles, but it is not yet clear that the changes caused by exposure to HUA are due to inhibition of proliferation or to some other effect of these drugs. This could include replication stress and DNA damage, for example (see point #2). Similarly, how do they know that "Mitotic activity shapes stage-specific histone modification profiles"? Again, this could be an effect of either drug that is independent of cell cycle. There are other instances throughout the manuscript. This concern can be addressed by modifying the text.

Answer:

We thank the reviewer for drawing our attention to these statements. To be more accurate in the description of our findings, we have rephrased them as follows:

Old title: “Mitotic activity shapes stage-specific histone modification profiles during *Xenopus* embryogenesis“

New title: “A systemic cell cycle block impacts stage-specific histone modification profiles during Xenopus embryogenesis”

Abstract, page 2, lines 19-20:

Old text: “These results indicate that replicational dilution of histone marks has a strong impact on developmental chromatin maturation.”

New text: “These results suggest that the cell cycle block by HUA alters stage-specific histone modification profiles.”

In addition, we have carefully revised the text in whole to avoid any over-interpretations.

The possible side effects of the inhibitors are dealt with below.

### Comment 2.

The authors should address (in the text) how they can distinguish whether the altered chromatin modification profile after HUA treatment is due to cell cycle arrest rather than a response to replication stress or DNA damage, which are associated with HU treatment. If they cannot rule these out, then they should at least discuss this limitation.

Answer:

A direct marker for DNA damage is γH2AX, which we do not find in our MS data. In addition, anti-γH2AX antibodies do not cross-react with the Xenopus protein. Neither are we aware of antibodies that recognize Xenopus RPA-S33Ph or CHK1-S345Ph, markers specifically associated with replicational stress. Therefore, we cannot directly assess how much replication stress and DNA damage is added by HUA treatment.

However, we have found the numbers of apoptotic cells to be roughly comparable between HUA and Mock embryos. A similar result has been obtained by Yeo & Gautier, 2003 [PMID: 12885553]), who incubated Xenopus gastrulae/neurulae with the same concentrations of HUA as we did, and tested for apoptosis by TUNEL-assay. Thus, the level of DNA damage caused by HUA appears to be sub-lethal for most cells. In addition, we have obtained ample evidence supporting the viability of HUA embryos, on which we rest our statement that the most likely cause for changes in histone modifications is indeed the cell cycle arrest.

Following the advice of the reviewer, we have added the following chapter to the revised discussion, which deals with these concerns (page 13, lines 20-29, and page 14, lines 1-14):

“In this study, we have addressed the impact of HUA-induced cell cycle arrest on the histone modification landscape during *X. laevis* embryogenesis. We assume that the observed changes in stage-specific histone modification profiles arise primarily in consequence to the cell cycle block. This assumption is based on multiple lines of independent evidence that testify to the viability of HUA-arrested embryos, such as intact embryonic patterning, correct spatiotemporal gene expression, survival to tadpole stage, low apoptotic rate, and last but not least, the reversibility of morphological, molecular and epigenetic phenotypes upon HUA-washout. But at this point, we cannot exclude that non-physiological effects of the inhibitors contribute to the observed chromatin changes. Specifically, hydroxyurea causes both replication stress and DNA damage through its breakdown products (Nazaretyan et al., 2018 [PMID: 29263100]; Ahmet Koç et al., 2004 [PMID: 14573610]). However, since DNA damage is associated with significant removal and degradation of core histones from DNA (Challa et al., 2021 [PMID: 33529595]; Nakamura et al., 2021[PMID: 33450211]), DNA damage and repair are unlikely to explain an increase in repressive histone PTMs, which are a hallmark of HUA chromatin. Finally, because Xenopus embryos develop by reductive proliferation, blocking the cell cycle leads to an increase in cell size. As recently shown, the ratio of surface area to volume of a cell determines the partitioning of IMPORTIN-a into cytosolic and plasma membrane-associated fractions, which scales intracellular structures, including nuclear size (Brownlee & Heald, 2019 [PMID: 30639102]). The relative availability of IMPORTIN-a for cytoplasmic-nuclear transport could influence the nuclear concentration of histone modifying enzymes -or factors regulating them - and thus affect histone PTM abundance. In conclusion, both physiological and non-physiological mechanisms may contribute to alterations in embryonic histone PTMs upon HUA treatment. In light of published evidence that S-phase dilution influences the propagation and maintenance of histone marks in cell lines (Alabert et al., 2015 [PMID: 25792596]; Alabert et al., 2020 [PMID: 31995760]), we consider the cell cycle block as a primary driver for the observed changes in histone PTM abundance.”

### Comment 3.

Very similar work addressing the effect of aphidicolin on morphogenesis was published by Rollins and Andrews in Development [PMID 1794324], and they came to similar conclusions about the impact of proliferation on differentiation during neurula and tailbud stages. This work should be discussed and cited in the introduction along with Harris and Hartenstein. The current manuscript makes a substantial advance beyond the 1991 Rollins paper, but it is an important and relevant background paper.

Answer:

We thank the reviewer for drawing our attention to the Rollins paper, which we were not aware of. The reference is now quoted together with Harris and Hartenstein in the introduction (page 4, lines 27-30).

### Comment 4.

Could the authors explain why they used both hydroxyurea and aphidicolin? Wouldn't aphidicolin be sufficient to inhibit proliferation, as in the Rollins paper?

Answer:

The combination of DNA synthesis inhibitors was chosen by us because “hydroxyurea alone works quickly (within 2hr), but does not inhibit synthesis completely, while aphidicolin works slower (within 4-6hr) but blocks DNA synthesis more completely” (quoted from Harris & Hartenstein, 1991 [PMID: 1901716], chapter: Experimental procedures/HUA treatment). This information is now provided in the first chapter of the Results (page 5, lines 15-16).

### Comment 5.

Fig 4B shows change in histone PTMs in HUA relative to control. I did not see an indication of how or whether they assessed the statistical significance of these changes. This should be included and mentioned either in the results section or the figure legend.

Answer:

The revised manuscript includes now statistical analysis of histone PTM abundance between the 4 time points (embryo developmental stages NF13, NF18, NF25, and NF32) of the control (Mock) and experimental (HUA) conditions. We performed Student’s t-test (paired, two tails). In Figure 4B, p-values smaller than 0.05 have been indicated by asterisks in the respective cells of the table. Full results of the statistical analysis are presented in the new panels B-D of Figure S3, and Supplementary Table 5 (S5 Table_MS Exp Type A). Additionally, we have changed the text in the Fig 4B legend and in Materials and methods section (page 22, lines 24-26, and page 23, lines 1-4).

### Minor comment 6.

On the first page of Results, it is not clear what is meant by "lies behind" in the sentence "The end point of the analysis (NF32) lies behind the phylotypic stage of Xenopus (NF28-31), when evolutionary conserved gene expression programs have established the vertebrate body plan." Do they mean the "end point" is after the phylotypic stage?

Answer:

Thank you for spotting an ambiguous wording. We meant to say that the “end point” is after the phylotypic stage. The revised sentence on page 6, lines 11-13 reads as follows:

“The endpoint of the analysis (NF32) is after the phylotypic stage of Xenopus (NF28-31), when evolutionary conserved gene expression programs have established the vertebrate body plan.”

### Minor comment 7.

The control to rule out developmental delay is important; they might consider moving to main figures. That's just a suggestion.

Answer:

Due to the small number of genes that were tested we have decided to leave this information in the Supplement.

Overall, this is interesting and important work.

We thank the reviewer for this supportive evaluation.

## Reviewer 2:

Pokrovsky et al. use mass spectrometry to analyse multiple histone modifications in developing Xenopus embryos and investigate the effect of permanently or temporarily withdrawing the cells from the cell cycle on changes in histone modification states. The study tackles a very interesting question: the extent to which passage through DNA replication and mitosis influences the histone modification status in cells, particularly during development. This is important because histone PTMs are thought to play an important role in generating and/or maintaining the gene expression program of differentiating cells. The paper is clearly written and the basic cell cycle and developmental parameters of the model are outlined well. The study potentially provides some useful data but is rather preliminary in its current form. It is not clear if the effects of hydroxyurea and aphidicolin can be ascribed only to withdrawal from the cell cycle. Effects such as replication stress and DNA damage responses, and also marked changes in cell size, could also be involved. Finally, it is not clear that the results have been analyzed in a statistically robust manner.

### Major point 1.

In Figures 4 and 6, the core results are provided as Z-scores. However, it is clear that all the scores are fairly low, with few exceeding +/-2, and are therefore are not very far from the center of the distribution. Figures S4 and S6 show the variability of the raw results, but it would be very useful to have some sense of the variability and statistical significance of the derived results (Z-scores and ratios) in the main Figures 4 and 6.

Answer:

The z-scores shown are based on row-wise (modification-wise) scaling of the data across all samples. These values are not suited for straight-forward statistical interpretation of group-wise differences. They simply serve to highlight differences in modifications across all samples. As the reviewer points out correctly, the differences are not massive. The new panels B-D in Figure S3 give an overview over the variability and statistical significance of the dataset. P-values for differences in histone PTM abundance between Mock and HUA conditions have been added to Supplementary Table 5 (S5 Table_MS Exp Type A).

### Major point 2.

Related to point 1, the absolute amount of H3S10ph peptides quantified appears very low (Fig S5 and S6). How does this affect the robustness of the findings? Are the results for low abundance marks generally less reliable than others? It is notable that another well-described mitotic marker, H3T3ph, shows the opposite pattern and apparently increases in HUA conditions. How can this be explained?

Answer:

For the sake of clarity we note that Figure S5 shows whole mount H3S10Ph immunostainings, while Figures S4 and S6 detail the relative abundance of this mark. Nevertheless, the question whether low abundant marks are less reliably quantitated than higher abundant ones, is important.

In order to evaluate the overall data distribution, and visualize the robustness of the findings, we have added an MA-plot (new panel B), as well as a Volcano-plot (new panel C) to Figure S3. As the reviewer correctly predicted, the graphs indicate that lower abundant modifications tend to have larger changes. Although not all fold-changes are statistically significant with our limited number of replicates, this dataset provides a large repository for Xenopus developmental biology and epigenetics fields, which includes useful information on many PTMs.

When we analyze H3S10Ph by immunostaining, both the decrease in the number of positive cells in arrested versus cycling embryos, as well as its recovery in Exp. B upon inhibitor washout, is statistically highly significant (p$<$0.001; n>3 biol. replicates) and testifies to the robustness of the cell cycle block and its reversibility.

Measured by mass spec in bulk embryonic chromatin, H3S10Ph is low-abundant (around 0.2%), because first, cells divide asynchronously, and second, mitosis takes up only a small fraction of time within the whole cell cycle (at NF32, for example, 15 min against 12 hours). This means that at the time an embryo gets fixed or lysed, only a small subset of cells carry this mark in physiological abundance, but the majority of cells does not. Despite the fact that H3S10Ph abundance is not significantly different between Mock and HUA, we notice that the drop in H3S10Ph is consistent and reproducible.

To make this result transparent in the revision, we have added inserts for H3S10Ph to Figures S4 and S6.

Mitotic chromatin is also enriched for H3T3Ph, and the reviewer noted that its abundance paradoxically appears to be higher in HUA, in potential conflict with our findings for H3S10Ph. We therefore scrutinized our Mass Spec data and performed additional experiments to investigate this point.

First of all, we confirmed that the H3T3Ph-peptide has been reliably identified and quantitated from the raw data (Figure I).

**Figure I.** Spectrograms of the MS measurements of the H3 aa 3-8 tryptic peptide with two isobaric histone modification states, T3Ph/K4p and K4p/T6Ph. The two isobaric forms are clearly separated as shown in the first (endogenous peptide) and second (heavy-labelled spike-in controls) panels. Endogenous K4p/T6Ph is not detected. There is no isotope interference within T3Ph/K4p (insert in the first panel). The correct identification was confirmed by matching the MS/MS fragmentation spectrum (fourth panel plus the insert).

Interestingly, we observed a large variability in its abundance between biological replicates. This is true for both Mock and HUA samples (see Figure II below).

**Figure II.** Representation of the relative H3T3Ph/K4p abundance in individual biological replicates for A) Mock and B) HUA conditions. Developmental stages indicated below. Values plotted are percentages of T3Ph/K4p among other coexisting modification states found within the H3 aa 3-8 tryptic peptide.

We suspected a technical problem behind this unusual variability and tested for miscleavage of the peptide H3 aa 1-8. We found that Trypsin cleavage at Arginine 2 is strongly impaired, when Threonine 3 is phosphorylated (see Figure III below).

**Figure III.** Extracted ion chromatograms (XICs) of the H3 aa 3-8 and 1-8 tryptic peptides. The first panel shows a full MS scan. When T3 is not phosphorylated, Trypsin cleaves H3 aa 1-8 peptide after R2 resulting in stable detection of H3 aa 3-8 peptide (H3T3un/K4p, second panel) and no detection of the uncleaved form (fourth panel). When T3 is phosphorylated, Trypsin cleavage after R2 is reduced (third panel), and peptide aa 1-8 (H3T3Ph/K4p) can be reliably detected on the MS1 level (fifth panel) and in the MS/MS fragmentation spectrum (sixth panel).

We then calculated the abundance of both un-phosphorylated and phosphorylated states of the miscleaved peptide H3 aa 1-8 and corrected the total abundance. This, however, did not reduce the variability between the biological replicates, but it slightly reduced the difference between Mock and HUA samples (see Figure IV below). We conclude that MS quantification of H3T3Ph levels had been correct.

**Figure IV.** Comparison of the H3T3Ph/K4p relative abundance without (A) and with (B) correction for the non-cleavage. Values plotted are average from 3 biological replicates percentages of T3Ph/K4p modification state among other coexisting modification states found within the H3 aa 3-8 tryptic peptide. T3Ph/K4p(-) = before correction, T3Ph/K4p(+) = after the correction. Error bars - s.e.m.

Due to the observed miscleavage at R2/T3Ph, we also implemented a correction for all other modifications found on the peptide H3 aa 1-8, i.e. K4p; K4me1/2/3; K4p/T6Ph, and K4me1/T6Ph. For all these modifications, except H3K4me3, the correction was in the range of 0.01-0.001% leading to no changes in numerical values or Log10-based colour codes. For H3K4me3, the correction affected the fold-change HUA/Mock in the HUAwo experiment. This altered the numerical value in Fig 6B from 1.64 to 1.63 (K4me3 second column). R2/T3Ph is the only case of trypsin miscleavage in our dataset.

We then decided to perform side-by side immunostainings for H3S10Ph and H3T3Ph on unprocessed embryos from the original series of Exp. A (Mock and HUA condition in pairs). The results are shown below in Figure V. Without exceptions, both marks were present in comparable numbers of cells in Mock embryos, and for both marks these numbers were diminished in HUA. Although these results are preliminary and not statistically validated, the immunostainings indicate that H3T3Ph-positive cell numbers become reduced by HUA condition.

**Figure V.** Comparative immunostainings for H3S10Ph and H3T3Ph marks.

Antibody-based detection methods have limits regarding specificity, sensitivity and linearity of signal. Moreover, the detection of histone PTMs is influenced by the state of chromosomal condensation. In contrast, quantification by Mass Spec is sensitive and specific and shows a broader linear range. Based on the results of the new experiments presented here, we are left to conclude that the H3T3Ph levels show an unexplained large variability in both Mock and HUA samples, which masks the mitotic enrichment of this modification.

In the revised manuscript, we have added the following statement (page 10, lines 11-16):

“Phosphorylation of Threonine 3 on histone H3 represents a second PTM that is mitotically enriched (Sawicka and Seiser, 2012 [PMID: 22564826]). For unknown reasons, this modification shows a large variability between biological replicates in our data set, resulting in a very similar averaged abundance for Mock and HUA conditions in all four stages (Table S5). Therefore, it cannot confirm our conclusions from the H3S10Ph mark.”

### Major point 3.

Also related to the above two points, how were the specific marks shown in Figures 4B and 6B selected? In the main, these are the most frequently discussed marks in the literature (eg H3K4me, H3K9me, H3K9ac, H3K27me), but I wonder if these are also the statistically most robust changes? Some notable marks such as H3K79me are ignored. Comparing the results of Mock B in Figure 6A with Mock A in Figure 4A there seem to be modifications which do not show such consistent changes in experiments A and B, though this is difficult to evaluate by eye.

Answer:

The selection of the histone PTMs in Figures 4B and 6B (relative ratio heatmaps) was solely based on established biological relevance for development and impact on gene activity. H3K79me was not selected, because in Xenopus Dot1L is dispensable during embryonic development, but becomes essential in pre-metamorphic tadpoles (Wen et al., 2015 [PMID: 25366346]). Therefore, our selection has been arguably biased in order to extract biological information from the observed trends. In consequence, the differences in selected histone PTMs are not always the most robust by statistical means. To indicate this, we have modified Figure 4B: now, the statistically different histone PTMs are marked with asterisks indicating p<0.05 (Student t-test, 2-tails, paired). Additionally, the full results of the statistical analysis are included in Supplementary Table 5 (S5 Table_MS Exp Type A).

The revised manuscript states this explicitly (page 10, lines 4-7):

“These PTMs were selected based on their biological relevance for embryonic development and impact on gene activity. PTMs, whose fold-change between Mock and HUA conditions is statistically significant (p<0.05), are marked in Figure 4B (and Table S5).”

The suspected inconsistency between data presented in Figure 4A and 6A, has a simple explanation: these two Figures cannot be compared directly. They represent different experiments (Exp.A versus Exp.B) and different types of values.

The heatmap in Figure 4A is based on the normalized absolute values of histone PTM abundance from 3 biological replicates of Exp. A. The heatmap in Figure 6A is based on the relative histone PTM abundance from a single replicate of Exp. B.

The two different experiments have been compared in Figure 6B, which displays the fold-change of selected histone PTMs. Overall, the results from the two experiments are similar. The full complement of relative histone PTM abundance for both experiments had been presented in Figure S6. We confirm this conclusion by the new Figure S3D, which shows that the overall fold-change of Exp. A is indistinguishable from the overall fold-change of Exp. B.

### Major point 4.

It is unclear how similar the clustering is in Figures 4A and 6A. If the results are robust, presumably these might be expected to be somewhat similar?

Answer:

Due to the differences in experimental set-up and the nature of data, the clustering in Figures 4A and 6A cannot be compared. Clustering in Figure 4A takes into account absolute values of the histone PTM abundance from four different developmental stages under two experimental conditions in triplicates. In contrast, Figure 6A displays relative histone PTM abundance from a single developmental stage and includes a new experimental condition (HUAwo) in one replicate. The reviewer's concern about the similarity between Exp. A and Exp. B is addressed in the new Figure S3D.

### Major point 5.

In Figure S1, I think there is confusion here between the inability to see a "statistically significant" difference between two results and the conclusion that this means there is no difference. For example, if I measure the height of 3 men and 3 women, I may very well conclude that there is no statistically significant difference. This does not mean that there is no difference in the average height of men and women. It simply means that the natural variation in height (coupled with measurement error) is too large for me to figure this out with a small sample size. What level of difference could have been detected with this assay given the variation observed? This is important so that we can evaluate the evidence that gene expression does not change.

Answer:

Thank you for spotting this incorrect interpretation of the statistical result. We have removed the sentence from Figure S1 legend.

### Major point 6.

I am not convinced that the HUA washout experiment allows us to "distinguish" "non-physiological derailment" from "the consequence of the G1/S arrest", and to "conclude that changes in the histone modification landscape arise as a consequence of the G1/S-phase cell cycle arrest". This experiment is certainly useful, but perhaps it could be introduced and interpreted more cautiously. It is quite possible that HUA causes non-physiological effects such as DNA damage and increased cell size, but that these are reversible. Confirming the results with an "orthogonal" approach to withdraw cells from the cell cycle would be very valuable.

Answer:

Upon re-reading our manuscript we became aware of the ambiguous meaning of our statement. Thinking of chromatin as a strongly interdependent ensemble of modifications, we were trying to convey the idea that if HUA-chromatin could be reversed by the inhibitor washout, then this could rescue stage-specific histone PTM profiles, instead of remaining altered as consequence of a toxic effect. We agree with the reviewer that becoming more normal upon HUAwo does not exclude side-effects of the drugs to be responsible for the initial perturbation, provided these side-effects are reversible. Also reviewer #1 has remarked on possible contributions from replication stress and DNA damage.

Consequently, we have written on page 11, lines 12-14 of our revision:

“To check whether the observed differences in morphology, gene expression, and histone modification landscape are reversible, we performed a transient cell cycle arrest (HUAwo) (Fig 1).”

In addition, we have obtained ample evidence supporting the viability of HUA embryos, on which we rest our statement that the most likely cause for changes in histone modifications is indeed the cell cycle arrest.

The notion that “HUA causes … increased cell size” deserves consideration. Xenopus embryos develop by reductive proliferation from the first cleavage until the start of feeding (i.e. days after the endpoint of our analysis). With every cell division, cells become smaller and smaller. Thus, blocking cell proliferation inevitably leads to larger cells. Because the cell volume dictates the scaling of cellular organelles, including nuclear size (Brownlee & Heald, 2019 [PMID: 30639102]), this may affect the epigenome.

Following the advice of the reviewer, we have added the following chapter to the revised discussion, which deals with these concerns (page 13, lines 20-29, and page 14, lines 1-14):

“In this study, we have addressed the impact of HUA-induced cell cycle arrest on the histone modification landscape during *X. laevis* embryogenesis. We assume that the observed changes in stage-specific histone modification profiles arise primarily in consequence to the cell cycle block. This assumption is based on multiple lines of independent evidence that testify to the viability of HUA-arrested embryos, such as intact embryonic patterning, correct spatiotemporal gene expression, survival to tadpole stage, low apoptotic rate, and last but not least, the reversibility of morphological, molecular and epigenetic phenotypes upon HUA-washout. But at this point, we cannot exclude that non-physiological effects of the inhibitors contribute to the observed chromatin changes. Specifically, hydroxyurea causes both replication stress and DNA damage through its breakdown products (Nazaretyan et al., 2018 [PMID: 29263100]; Ahmet Koç et al., 2004 [PMID: 14573610]). However, since DNA damage is associated with significant removal and degradation of core histones from DNA (Challa et al., 2021 [PMID: 33529595]; Nakamura et al., 2021[PMID: 33450211]), DNA damage and repair are unlikely to explain an increase in repressive histone PTMs, which are a hallmark of HUA chromatin. Finally, because Xenopus embryos develop by reductive proliferation, blocking the cell cycle leads to an increase in cell size. As recently shown, the ratio of surface area to volume of a cell determines the partitioning of IMPORTIN-a into cytosolic and plasma membrane-associated fractions, which scales intracellular structures, including nuclear size (Brownlee & Heald, 2019 [PMID: 30639102]). The relative availability of IMPORTIN-a for cytoplasmic-nuclear transport could influence the nuclear concentration of histone modifying enzymes -or factors regulating them - and thus affect histone PTM abundance. In conclusion, both physiological and non-physiological mechanisms may contribute to alterations in embryonic histone PTMs upon HUA treatment. In light of published evidence that S-phase dilution influences the propagation and maintenance of histone marks in cell lines (Alabert et al., 2015 [PMID: 25792596]; Alabert et al., 2020 [PMID: 31995760]), we consider the cell cycle block as a primary driver for the observed changes in histone PTM abundance.”

We agree that an orthogonal approach would be helpful to confirm our results, however, we are unaware of experimental means that are equally suited as HUA to block the cell cycle for a comparable duration of time, being compatible with terminal differentiation, and have less side effects. As stated by Harris and Hartenstein, splitting up Hydroxyurea and Aphidicolin into separate treatments is not advisable. However, determining the genome-wide deposition of histone marks, whose abundance is altered by HUA treatment, provides a strategy to evaluate the impact of a cell cycle block on the developmental epigenetic landscape in future experiments.

### Minor point 1.

There is a standard nomenclature for histone modifications, and it is not clear why there are inconsistent deviations from this in the paper. For example, methylation is sometimes "me" (the standard) and sometimes "m", sometimes "Me", and even occasionally "met" (Table S2). Standard notation for phosphorylation is "ph", but is typically "Ph" in this paper, and even "p" in Table S2. This latter usage is particularly confusing because "p" is used throughout the other figures and tables in a way that is not defined, but is evidently not for phosphorylation. Does this "p" refer to propionylation? If so, it should be prominently defined in the text.

Answer:

We thank the reviewer for spotting this inconsistency. We have corrected it throughout the manuscript, figures, figure legends, and tables. In general, we decided to stick to the classical abbreviations for methylation - “me”, and acetylation - “ac”. To avoid misunderstandings and ease the reading we decided to distinguish propionylation and phosphorylation by indicating them as “p” and “Ph” respectively. The proper nomenclature is now defined and added in the corresponding figure legends.

### Minor point 2.

Propionylation seems an important step in this process, but the methods do not seem to mention it.

Answer:

Propionylation is indeed a crucial step during histone sample preparation for the mass spectrometry analysis. It had been mentioned in the Materials and methods section of the original manuscript, which is called “Histone acid extraction and sample preparation” (... Histone bands were excised and propionylated as described before [[64]](https://paperpile.com/c/UdkJ31/7h3Cf).)

In the revised manuscript, we have added the following sentence to the Materials & Methods section (page20, lines 22-28) and to the legend of Figure S7:

“Propionylation blocks all endogenously unmodified and monomethylated Lysine residues from being cleaved in the subsequent trypsin digest, thereby creating an optimized peptide pool for mass spectrometry analysis. Due to this step naturally unmodified Lysine residues are labelled as “p”, tryptic peptides which have no modification states indicated as “un”. Arginine10-labelled forms of the PTMs of interest (500fmols/peptide, JPT Peptide Technologies) were added to the gel samples together with trypsin.“

### Minor point 3.

Line colors do not match the key in Fig S1A.

Answer:

The colours have been matched between the colour key and lines in Figure S1A.

### Minor point 4.

In the Methods section on Heatmap Generation, it mentions n = 3, but this is only true for Figure 4A. For Figure 6A, n= 1.

Answer:

That there is only 1 replicate for the HUAwo in Exp. B had been indicated in the legend of Fig 6A, however, the Methods section was misleading.

We have added the following sentence to the chapter “Heat map Generation” (page 23, lines 17-19): “For heatmap generation and hierarchical clustering in Fig 6A values of relative histone PTM abundance were used (n=1 biological replicate at developmental stage NF32).“
