# Supplementary material for: A systemic cell cycle block impacts stage-specific histone modification profiles during Xenopus embryogenesis
Source: PLoS Biol. 2021 Sep 7;19(9):e3001377. doi: 10.1371/journal.pbio.3001377 (PMC8535184; doi:10.1371/journal.pbio.3001377)
Supplement: Attachment — Submitted filename: Response to the reviewer.docx [file pbio.3001377.s025.docx]

Reviewer’s comment:

The authors have done a very good job adjusting the text to reflect the points made by the reviewers. In particular, they have modulated the language to better reflect the results. I do have a substantial remaining concern however. Usefully, the authors have now provided some statistical analysis of their data. For this, they use paired Student t tests. However, no account is taken of the fact that they are making multiple comparisons. This is very important here, and I do not believe the manuscript should be published in its current form. For example, in Figure 4B, they make 88 comparisons (and actually many more if you take the whole dataset into account - see Supplemental Tables), but use unadjusted p values. Essentially, they have to take into account the principle that it more likely that you'll get a head on a coin if you throw it multiple times than if you throw it once. Whether publication is then warranted would depend, of course, on the extent to which they then convincingly show statistically significant changes in repressive histone modifications. The fact that the magnitude of the changes they observe are, as they admit, small, reinforces the importance of this analysis.

Answer:

We agree with the reviewer that multiple testing correction procedure is needed. We have revised our manuscript accordingly.

Following the concerns about the multiple comparisons and the resulting inflation of type I error rates, we have now implemented the Benjamini-Hochberg procedure, which corrects for multiple testing by controlling the false discovery rate (FDR). The corresponding changes were implemented in Figures 4B, 6B, S3B and C, and in the corresponding figure legends. Additionally, we added adjusted p-values in the supplementary Table S5 for the entire dataset, not only for the selected histone PTMs as shown in Fig 4B and 6B. Indeed, the results do support our conclusion that an experimentally induced block in the cell cycle leaves an imprint on the epigenetic landscape.

The manuscript has been changed in the following places (revised text in blue):

Page 7, line 15: “One quarter of the analysed histone PTMs is significantly different between Mock and HUA conditions (Fig S3C and Table S5).”

Page 7, lines 20-22: “PTMs, whose fold-change between Mock and HUA conditions is statistically significant (adj.p<0.1, Benjamini-Hochberg procedure), are marked in Figure 4B (and Table S5).”

Page 16, lines 42-43: “In case of multiple testing, the Benjamini-Hochberg FDR procedure was performed - indicated as “adj.p”.”

We also changed the figure legends:

Figure 4B: “Asterisks (*) - adj.p<0.1 (Benjamini-Hochberg procedure).”

Figure 6B: “Asterisks (*) - adj.p<0.1 (Benjamini-Hochberg procedure) between indicated conditions.”

Figure S3B: “Histone PTMs different between HUA and Mock conditions with adj.p<0.1 (Benjamini-Hochberg procedure) highlighted in red. One quarter of the analysed histone PTMs is significantly different between Mock and HUA conditions.”

Figure S3C: “Volcano plot shows the distribution of Log2 transformed fold-change [Log2(fold-change)] over negative Log2 transformed adj.p-value [-Log2(adj.p)] between HUA and Mock conditions (Exp. A). Baseline in grey: 3.3219 as -Log2(0.1). Histone PTMs different between HUA and Mock with adj.p<0.1 (Benjamini-Hochberg procedure) are above the baseline, highlighted in red. One quarter of the analysed histone PTMs is significantly different between Mock and HUA conditions.”

Importantly, repressive histone PTMs (H3K9me3, H3K27me3, H4K20me3) remain significantly different between HUA and Mock conditions after the FDR adjustment.
