# Supplementary material for: A systemic cell cycle block impacts stage-specific histone modification profiles during Xenopus embryogenesis
Source: PLoS Biol. 2021 Sep 7;19(9):e3001377. doi: 10.1371/journal.pbio.3001377 (PMC8535184; doi:10.1371/journal.pbio.3001377)
Supplement: Attachment — Submitted filename: Response to the reviewer.docx [file pbio.3001377.s026.docx]

Reviewer’s comment

Rev. 2:

The authors have carried out multiple comparison adjustments as requested. The significance of most of the results fail to reach the typically used cut off of p < 0.05, which itself is not very stringent, and the authors instead use p < 0.1. This means that the relatively low fold-changes seen in this work are also not in general of huge statistical significance. Ideally, the paper should provide an explanation of why this atypical significance cut-off is used, together with a clear discussion of how the results should be interpreted. I think the validity of changes in any single histone modification in this analysis has to be considered very cautiously (for any given mark, the change seen could be observed in up to 1 in 10 experiments even on the null hypothesis), but the broader conclusion that some histone marks in general do change is probably reasonable.

Answer:

It is important to distinguish group level error assessment from individual error assessment. The FDR (https://www.pnas.org/content/100/16/9440) provides control on the fraction of false positives in a set of significant results on multiple testing studies. A threshold applied on the FDR therefore does not say anything about the false positive rate of the individual test as suggested by the reviewer.

Furthermore, while the 5% significance level alpha applied to individual tests is due to a common agreement, thresholds for the adjusted p-value (FDR) are set more flexibly. Some commonly applied procedures, such as DESeq2 for differential gene expression assessment, have a default cut-off of 10%. This is the threshold, which we have applied in our manuscript. It certainly does not represent an “atypical significance cut-off”.

We agree with the reviewer that any conclusions on individual modifications should be treated with caution, not only due to a low statistical significance of the reported changes, but also due to the cellular heterogeneity of the embryo, whose bulk chromatin provides the average abundance of individual modification states, but not their abundance (or genome-wide distribution) in single cell types.

This has been acknowledged in a new paragraph found on page 10 line 42 to page 11 line 5:

“For an exploratory study like this, the limited replication negatively impacts the statistical interpretation of low abundant PTMs exerting high variance, such as H3S10Ph (see Fig 2, Data S1, Fig 4B, Table S5, Data S6), despite the large mean fold-change. On the contrary, for highly abundant modifications with very low variance, such as unmethylated H3 Lysine 4 (H3K4p), much smaller effects appear statistically significant, while being most likely biologically irrelevant (Fig 4B, Table S5). Therefore, the statistical results for any individual modification need to be interpreted with caution. While keeping this in mind, we focus our further discussion on the magnitude of the observed fold-changes for biologically relevant PTMs involved in gene regulation. We hope that the observed trends will stimulate independent experiments in other systems.”
